# A case with febrile attacks and vasculopathy associated with ADA2 and MEFV gene mutations

**DOI:** 10.1186/1546-0096-13-S1-P9

**Published:** 2015-09-28

**Authors:** H Ozdogan, S Ugurlu, A Hacioglu, E Tahir Turanli, A Kirectepe Aydin

**Affiliations:** 1 Cerrahpasa Medical Faculty, University of Istanbul, Division of Rheumatology, Department of Internal Medicine, Istanbul, Turkey; 2 Istanbul Technical University, Molecular Biology and Genetics Department, Science and Letters Faculty, Istanbul, Turkey; 3 Istanbul Technical University, Dr Orhan Öcalgiray Molecular Biology-Biotechnology and Genetics Research Centre, Istanbul, Turkey

## Background

Decreased activity of ADA2, caused by recessive mutations in CECR1 gene (Cat Eye Syndrome Chromosome Region, Candidate 1 also known as ADA2), results in cutaneous or systemic vasculitis with variable clinical manifestations.

## Case

A patient with juvenile onset recurrent febrile attacks associated with familial polyarteritis nodosa (PAN) and who carries CECR1 and MEFVgene mutations is described. The index case, a 23 year old male patient with recurrent attacks of fever and arthritis since the age of 7 was diagnosed initially as Familial Mediterranean Fever (FMF). A beneficial response to treatment with colchicine was observed. A year later he developed livedo reticularis and nodular dermal lesions compatible with cutaneous PAN. He was treated with prednisolone and azathioprine. Arthralgia, fever and dermal lesions regressed and he was in remission until he developed anemia and macrocytosis a year later and azathioprine was stopped. Due to the activation of his skin vasculitis anakinra 100mg/day was instituted. The beneficial response obtained with anakinra was lost when he discontinued the treatment. A family history revealed a brother two years older than himself who also had livedo reticularis, Raynaud's phenomena, fever and arthritis since the age of 8, diagnosed as PAN before the index case and died at age 22 because of gut perforation secondary to acute mesenteric ischemia. With the probable diagnosis of ADA2, the index patient was analyzed for CECR1 gene mutations on chromosome 22q11.1. After amplification of the exons 2, 4, 5, 6 and 9 on PCR, DNA sequencing analysis was performed. A homozygous c.139G-A transition in exon 2, resulting in a gly47-to-arg (G47R) substitution at a highly conserved residue in the dimerization domain was identified in this patient who was known to be heterozygous for M694V and R202Q mutations of the MEFV gene. His vasculitic lesions responded to Infliximab after the fourth infusion together with daily colchicine treatment.

## Conclusion

CECR1 gene mutation should be considered in cases presenting especially with early onset PAN. Infliximab maybe an effective therapy in these cases which are related with increased mortality. This is the first case that is reported to carry both CECR1 and MEFV gene mutations presenting with characteristic phenotypes of FMF and ADA2. The role of CECR1 mutations in the well documented association of FMF and PAN will be an interesting field of investigation in near future.

## Consent to publish

Written informated consent for publication of their clinical details was obtained from the patient/parent/guardian/relative of the patient.

